# Complete Resolution of Pseudomalignant Erosion in a Reflux Gastroesophageal Polyp with Proton Pump Inhibitor

**DOI:** 10.1155/2015/657059

**Published:** 2015-11-24

**Authors:** Takahiko Nakajima, Haruo Yagi, Hayato Baba, Takashi Minamisaka, Shigeharu Miwa, Shinichi Hayashi, Takeshi Nishida, Hideki Hatta, Koichi Tsuneyama, Johji Imura

**Affiliations:** ^1^Department of Diagnostic Pathology, Graduate School of Medicine and Pharmaceutical Science, University of Toyama, 2630 Sugitani, Toyama 930-0194, Japan; ^2^Department of Surgery, Kouseiren Namerikawa Hospital, 119 Tokiwa-cho, Namerikawa 936-8585, Japan; ^3^Department of Molecular and Environmental Pathology, Institute of Health Biosciences, University of Tokushima Graduate School, 3-8-15 Kuramotomachi, Tokushima 770-8503, Japan

## Abstract

Pseudomalignant erosion is a diagnostic pitfall for pathologists in the differential diagnosis of malignant neoplasms. Here, we present a challenging case of a biopsy specimen from the eroded head of a polyp at the esophagogastric junction. A malignant neoplasm could not be ruled out due to the presence of bizarre stromal cells. A second biopsy performed after the administration of a proton pump inhibitor (PPI) for 4 weeks revealed endoscopic resolution of the polyp along with the complete histological resolution of the bizarre stromal cells and led to the diagnosis of pseudomalignant erosion in a reflux gastroesophageal polyp. In conclusion, histological and endoscopic response to PPI therapy is an important clue for the correct diagnosis of reflux gastroesophageal polyps with pseudomalignant erosion.

## 1. Introduction

Reflux gastroesophageal polyp is an inflammatory polypoid lesion at the esophagogastric junction caused by reflux esophagitis [1–3]. The histology of the polyp shows hyperplastic cardiac mucosa with or without squamous epithelium. The surface of the polyp is often eroded, and the stroma is replaced by inflammatory granulation tissue.

Atypical stromal cells with bizarre nuclei, which resemble malignant cells, may sometimes be found in gastrointestinal erosions as a reactive process. Importantly, the presence of atypical stromal cells in biopsy specimens may lead to misinterpretation of the lesion as malignant; thus, these lesions are called pseudomalignant erosions [1, 4]. When reflux gastroesophageal polyps are accompanied with pseudomalignant erosion, biopsy specimens obtained from the polyps confound the pathologist in reaching a correct histological diagnosis, particularly in cases with striking atypia. Even though benign in nature, these polyps can rapidly grow in size and are mostly resected through endoscopy due to the suspicion of malignancy [2, 3].

We herein present a challenging biopsy case of a polyp at the esophagogastric junction with bizarre stromal cells. A malignant neoplasm could not be completely ruled out at initial disease presentation; however, a definitive diagnosis of pseudomalignant erosion in a reflux gastroesophageal polyp was made upon the resolution of the polyp with proton pump inhibitor (PPI) therapy. We discuss the pitfalls and the use of clinical response to the PPI therapy in the diagnosis of reflux gastroesophageal polyp.

## 2. Case Presentation

**Figure 1:**
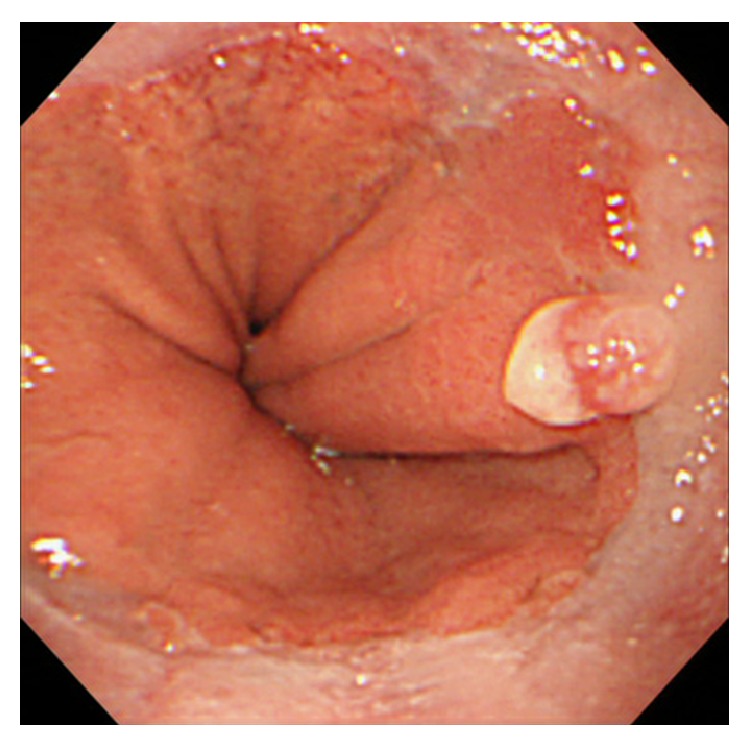
Endoscopic view of a polyp at the esophagogastric junction at the initial disease presentation.

**Figure 2:**
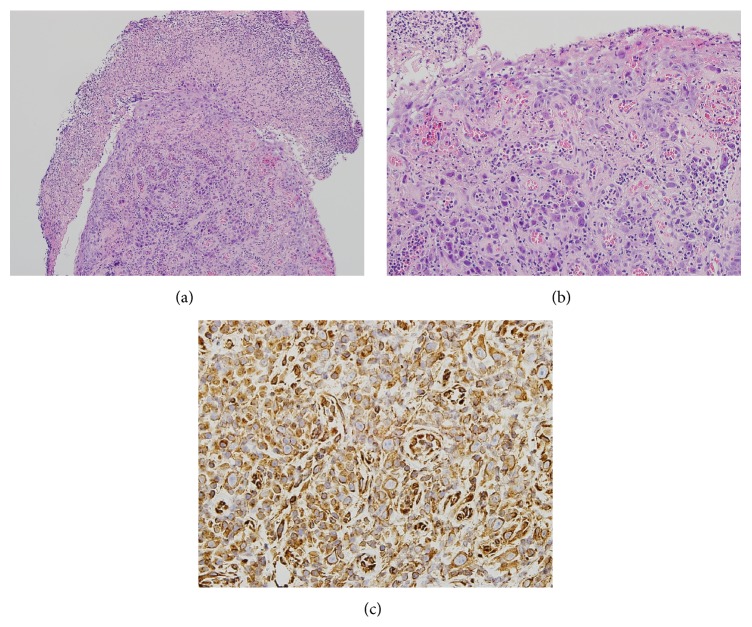
Light microscopic findings. ((a) and (b)) Bizarre stromal cells with large, hyperchromatic, atypical nuclei containing prominent nucleoli are observed using hematoxylin and eosin (HE). (c) The atypical cells were immunopositive for vimentin. (a) Original magnification 100x, (b) original magnification 400x, and (c) original magnification 400x.

A healthy, 62-year-old, asymptomatic male underwent upper endoscopy as part of a routine checkup. A small, semispherical polyp was detected at the esophagogastric junction (Figure 1). Mucinous exudate and erosion on top of the polyp were observed. Microscopic findings of the biopsy specimen taken from the polyp showed bizarre cells with large, hyperchromatic, atypical nuclei containing prominent nucleoli that were scattered beneath the surface squamous epithelium and the eroded surface (Figures 2(a) and 2(b); stain: hematoxylin and eosin; original magnification: 100x and 400x, resp.). The histopathological differential diagnoses of these findings included pseudomalignant erosion, sarcoma, malignant lymphoma, amelanotic melanoma, and viral infection. Immunohistochemistry showed atypical, subepithelial cells positive for vimentin and negative for leukocyte common antigen, cytokeratins, smooth muscle actin, HHF35, CD68, S100, and cytomegalovirus antibody; these results suggested sarcoma with muscle or histiocytic differentiation, malignant lymphoma, malignant melanoma, and viral infection as unlikely for diagnosis (Figure 2(c); stain: immunohistochemistry with anti-vimentin antibody; original magnification: 400x). Yet, it was not possible to histopathologically distinguish the pseudomalignant erosion from sarcomas of other lineages.

**Figure 3:**
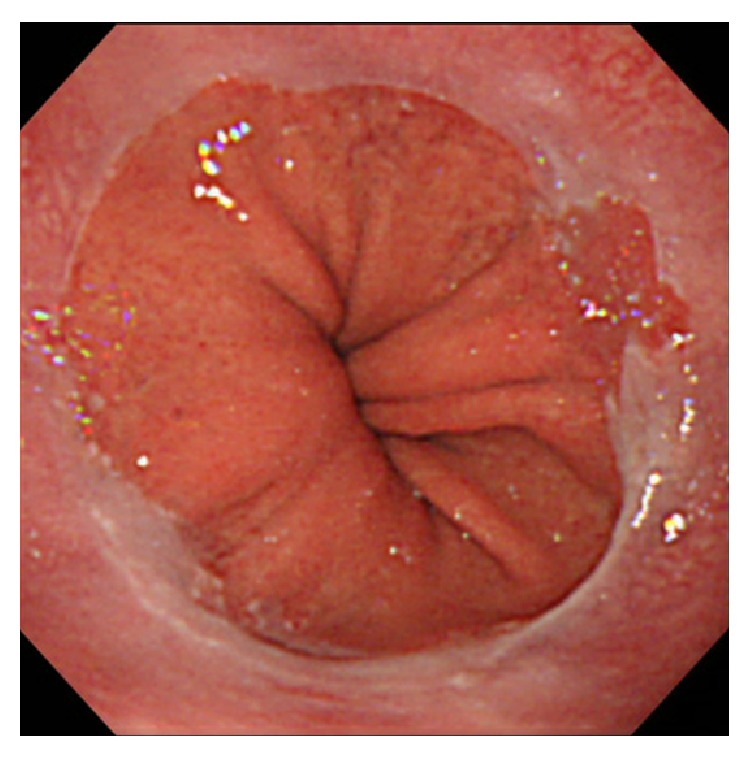
Endoscopic view after 4 weeks of the administration of a proton pump inhibitor. Note the complete disappearance of the polyp.

In previous studies, PPI therapy was shown to be effective in removing these polyps; hence, we used this procedure. A second endoscopy was performed after administration of a PPI for 4 weeks, and no polyps were detected (Figure 3). The biopsy specimen from the same site revealed squamous and cardiac mucosa without atypical changes. The clinical response to the PPI therapy allowed for a diagnosis of pseudomalignant erosion in the reflux gastroesophageal polyp to be made.

## 3. Discussion

Pseudomalignant erosion in the gastrointestinal tract has been noted in association with polyps and ulcers [1]. The bizarre stromal cells, thought to be of fibroblastic origin, are formed as a result of chronic irritation and ulceration in the underlying benign lesions [5, 6]. In the esophagus, a reflux gastroesophageal polyp is the common underlying lesion of pseudomalignant erosion [1] and is considered to result from the mucosal regenerative response to the reflux esophagitis-mediated mucosal injury. Thus, the biopsy specimens taken from the eroded surface of the polyp may be misinterpreted as a malignant neoplasm.

It is crucial to distinguish pseudomalignant erosion from sarcoma as surgical intervention is required for the latter. The endoscopic resection used to be the treatment of choice for reflux gastroesophageal polyps due to the suspicion of malignancy. However, with the clinical choice of PPI therapy, the complete resolution, or at the least a substantial decrease in size, of the reflux gastroesophageal polyps is expected [7–9]. To avoid unnecessary surgical resection, clinical response to PPI therapy should be considered to reach a correct diagnosis of pseudomalignant erosion in a reflux gastroesophageal polyp.
